# Hydrogen Bond Dynamic Propensity Studies for Protein Binding and Drug Design

**DOI:** 10.1371/journal.pone.0165767

**Published:** 2016-10-28

**Authors:** Cintia A. Menéndez, Sebastián R. Accordino, Darío C. Gerbino, Gustavo A. Appignanesi

**Affiliations:** INQUISUR-UNS-CONICET and Departamento de Química, Universidad Nacional del Sur, Bahía Blanca, Argentina; Wake Forest University, UNITED STATES

## Abstract

We study the dynamic propensity of the backbone hydrogen bonds of the protein MDM2 (the natural regulator of the tumor suppressor p53) in order to determine its binding properties. This approach is fostered by the observation that certain backbone hydrogen bonds at the p53-binding site exhibit a dynamical propensity in simulations that differs markedly form their state-value (that is, formed/not formed) in the PDB structure of the apo protein. To this end, we conduct a series of hydrogen bond propensity calculations in different contexts: 1) computational alanine-scanning studies of the MDM2-p53 interface; 2) the formation of the complex of MDM2 with the disruptive small molecule Nutlin-3a (dissecting the contribution of the different molecular fragments) and 3) the binding of a series of small molecules (drugs) with different affinities for MDM2. Thus, the relevance of the hydrogen bond propensity analysis for protein binding studies and as a useful tool to complement existing methods for drug design and optimization will be made evident.

## Introduction

The hydration shell of a protein is known to present heterogeneous features, particularly at binding sites [1–10]. In fact, the main role of hydration of binding sites has been assessed in protein-protein association and ligand binding [1–10]. Specifically, labile (or “unfavorable'') hydration-water molecules are expected to be replaced by groups of the ligand complementary to the protein surface, thus driving the binding process [1–4]. Indeed, computational structure-based strategies [2–4] have been successful in exploiting this knowledge. In turn, it is well-known that backbone hydrogen bonds (BHBs) represent main interactions responsible for shaping protein secondary and tertiary structural motifs. In solution, such non-covalent interactions are protected from the disrupting effect of water by the protein aminoacid side-chains. However, at the binding sites of apo proteins, BHBs might not be properly stabilized but solvent-exposed [1–10]. In fact, such packing defects (incompletely wrapped BHBs named “dehydrons” [6,7] have been extensively described in the literature [6,7] together with their relevance for protein association and binding [6–10]. Dehydrons represent non-covalent interactions (BHBs) with an increased dehydration propensity since water removal upon ligand association results in a stability gain [6–10]. Indeed, hydration water has been shown to be labile at such motifs [10]. These structural packing defects are straightforwardly determined from the Protein Data Bank (PDB) structures of apo proteins, thus fostering novel avenues for drug design [6–10].

As we have described in detail previously [11], the above expounded scenario is mainly rooted on structural facts while proteins are inherently dynamical objects and, thus, the structural information of the PDB, with the corresponding non-covalent interactions, might be veiling valuable information regarding protein interactions and function. In fact, we have shown that certain BHBs of apo proteins present a dynamic propensity (when studied in simulations that start form the PDB structure of the protein) that markedly differ from their corresponding PDB state-value [11]. That is, a few BHBs that are formed in the PDB of the apo protein display a conspicuous disruption tendency in the dynamics while, on the contrary, other BHBs are prominently formed in the protein dynamics while they are not established in the PDB structure [11]. We have also shown that protein binding sites are enriched in such “chameleonic'' BHBs (CBHBs, since they change state from the PDB prescription to the opposite formation propensity in solution), we have connected them to the binding-site hydration properties by means of water density fluctuation calculations (which estimate the work implied in water removal) and uncovered their role as drug targets [11]. This result puts the spotlight on chain dynamics and reveals backbone hydrogen bond dynamic propensity as a relevant tool for protein binding studies and drug design/optimization. Within this context, in the present work we shall employ this conceptual framework to rationalize the results of computational alanine-scanning studies for a system of great interest, the complex of the tumor suppressor p53 (a molecule that is found to be mutated in half the cases of cancer [12–18]) with its natural regulator, the protein MDM2. We shall also explain the binding to MDM2 of a small molecule (Nutlin-3a [19, 20], a disruptor of the MDM2-p53 interface), also dissecting the contribution of the different substituent groups of the molecule. Finally, we shall study the binding of a series of small molecules (drugs) with different affinities for MDM2 to make evident the relevance of this new concept to assist traditional methods in drug design efforts.

## Materials and Methods

### Molecular dynamics simulations

In this work we shall focus on the interaction between the tumor suppressor p53 and the protein MDM2 [19,20]. The PDB entry of this complex is PDB: 1YCR, while for the apo MDM2 protein is PDB:1Z1M. In fact, as in all the simulations in this work, we simulate the apo form of the N-terminal domain of MDM2, MDM2^N^, and a complex of MDM2^N^ with a peptide fragment taken from the transactivation domain of p53 [21]. We shall also study the interaction of MDM2 with the disruptive molecule Nutlin-3a (PDB: 4HG7) and with other small molecules: compound 14-b (PDB: 3VZV) [22], compound 1-a [23] (PDB: 3JZK) and compound Ding-1a [24]. However, our analysis is generally applicable to the protein-protein and drug-protein binding contexts. To study the dynamical behavior of these systems, as described in more detail in a previous work [11], were carried out molecular dynamics simulations by means of AMBER simulation package 14 [25], using in all cases periodic boundary conditions, TIP3P water and T = 300 K, always with the same minimization and equilibration protocol (equilibration was tested by monitoring the behavior of thermodynamical properties like temperature, pressure and energy oscillations). In the case of the MDM2-p53 complex (for the studies of the wild type complex and the computational alanine scanning studies) we performed production runs of 40 ns with 2 fs time step, recording 8000 equally spaced configurations. For the MDM2- apo protein we performed production runs of 50 ns with 2 fs time step, recording 10000 equally spaced configurations. For all other systems production dynamics were performed for 20 ns with 2 fs time step and saving 4000 configurations. Again as described in more detail in our previous work [11], we used a simple geometrical method to determine the binding site of the MDM2 protein by finding BHBs in the protein whose distance (measured form the N amide or the carbonyl O) to any heavy atom of the p53 protein in the MDM2/p53 complex (PDB: 1YCR) is less than 6 Å [9,10,11]. This method yields BHBs located within a certain interface or binding site of interest, while other interacting/binding sites of the protein might also exist.

### Dynamic analysis of BHB propensity

Based on the data from the PDB: 1Z1M of the apo MDM2 protein we built a backbone hydrogen bond contact matrix (PDB-BHB-CM) following the method introduced in our previous work [11], where each matrix element {i, j} is either 1 or zero, provided the corresponding pair of residues i and j satisfy a hydrogen bonding criterion (N-O cutoff distance, r < 3.5 Å; N-H-O cutoff angle, θ > 140° [10]) or not, respectively. We next recorded a time-averaged, or dynamic, BHB contact matrix (DBHB-CM) [11]. This was done by calculating the fraction of time each BHB is formed during long runs after equilibration [11]: At each evaluation time, if a pair of residues i and j satisfy the hydrogen bonding criterion, the corresponding {i, j} matrix element becomes 1, while it is 0 otherwise. Then, we averaged the results for each matrix element at all evaluation times (in fact, instead of the HB criterion indicated, a formation criterion based solely on the evaluation of the N-O distance for all times would suffice for this matter). Thus, the DBHB-CM contains fractional values that range from 0 (never formed) to 1 (formed all the time) for the different matrix elements [11].

Again following the method we described in more detail in [11], and to contrast the dynamical behavior of the protein with the information provided by the PDB structure, we calculated, for each matrix element of the DBHB-CM, its (absolute value) distance, D, with respect to the corresponding matrix element of the PDB-BHB-CM [11]. This means that for each BHB we calculated the difference between its dynamics formation propensity (its time-averaged value during the dynamics) and its corresponding PDB-structure state-value. By analyzing the D values for each matrix element we find that most BHBs of the PDB are stable during the dynamics (low distance value, D). However, certain BHBs exhibit large D-values. As we have already shown [11], these cases correspond to interactions that are present in the PDB and disappear during the dynamics or, else, to interactions that, while absent in the PDB are nonetheless persistently formed during the dynamics. If the absolute value of D for a given BHB is above ½, we call it a “chameleonic” BHB, CBHB [11], since such BHB “changes it state'' (from formed to mostly disrupted or from not formed to mostly formed) between the PDB structure and the dynamics. Thus, as we have already noted [11], the actual dynamical state in solution of such interaction is “hidden'' in the PDB structure of the apo protein under an opposite state-value (which might thus confer a misleading dynamical expectation). Additionally, when we study the location of such CBHBs, we find a clear enrichment at the p53 binding site of MDM2, thus pointing to their relevance for the binding process. In what follows we focus on CBHBs located at the binding site and, for simplicity, we shall term them as C-HBs. The C-HBs at the p53 binding site of MDM2 are the following: LEU 57-VAL 53 **(D = -0.9**), MET 62- GLY 58 (**0.69**), ILE 99- GLU 95 (**0.56**), TYR 100- HIS 96 (**0.46**) and ILE 103- TYR 99 (**0.84**). Thus, the BHB LEU 57-VAL 53 is not formed in the PDB structure (state value of zero) of the apo MDM2 while we find it to be formed in 90% of the simulation time (a formation propensity of 0.9). Thus, the value of D is negative, D = 0–0.9 = -0.9. In the other cases, we are dealing with BHBs that are established in the PDB (state value equal unity) while they display a high formation tendency in the dynamics. For example, MET 62- GLY 58 has a state value of 1 in the PDB while it is found to be formed only 31% of the time in the dynamics. Thus, D = 1–0.31 = 0.69. We regard a BHB as a C-HB if the absolute value of D is large than 0.5 (this value is arbitrary it but can be justified by the existence of a neat bimodality in the distribution of this parameter for a set of proteins, with a minimum value around 0.5). However, here we also consider the BHB TYR 100- HIS 96 as a C-HB given the proximity of its D-value to 0.5.

## Results and Discussion

### Formation propensity of C-HBs in the MDM2-p53 complex

Having identified the C-HBs of the apo MDM2 protein, we now estimate the stability of such hydrogen bonds in the complex MDM2 forms with p53 (PDB: 1YCR). To this end, we calculated the fraction of time these interactions are disrupted during long simulation runs of 40 ns. In other words, for the MDM2-p52 complex we calculate the D-values, since for each C-HB the fraction of time broken is just the difference between the state value in the PDB of the complex (equal to unity given the fact that these BHBs are always formed in the PDB) and the fraction of time they are formed in the dynamics (thus, at variance form the situation for the apo protein, all D-values are positive for the complex p53-MDM2). The results are shown in Table 1:

**Table 1:** Hydrogen bond formation propensity for the C-HBs of MDM2, both in the apo protein and in the p53-MDM2 wild-type complex. In both cases, we denote as PDB-BHB the state value of the BHB in the PDB (1 = formed; 0 = not formed), D-BHB is the fraction of time the BHB is formed in the dynamics and the value D is given by: D = PDB-BHB—D-BHB. We note there that the C-HBs are determined in the apo protein and they are named as such also when considered in the complex. From their behavior in the protein complex we would not identify them as C-HBs. Thus, C-BHs are “quenched” upon association.

C HBs		MDM2 apo protein			MDM2-p53 complex		
		PDB-BHB	D-BHB	D	PDB-BHB	D-BHB	D
57	53	0	0.9007	-0.9007	1	0.9275	0.0725
62	58	1	0.3081	0.6919	1	0.94325	0.05675
99	95	1	0.4368	0.5632	1	0.82825	0.17175
100	96	1	0.5519	0.44809997	1	0.904	0.096
103	99	1	0.1549	0.8451	1	0.8375	0.1625

From such results we can learn that, at variance form the situation in the apo form of MDM2, all the C-HBs are now strongly stabilized in the complex with p53. All of them (including the BHB 57–53) are formed in the PDB of the complex and remain formed most of the time in the simulation of such complex, thus yielding very low D-values. Hence, we can easily learn from this behavior that such non-covalent interactions clearly depend on intermolecular stabilization and, thus, they promote the association process. In this sense, this C-HB quenching effect might be exploited as a novel design concept for protein binding and drug design.

### Computational alanine scanning study of the MDM2-p53 interface

We next performed a computational alanine scanning study of the MDM2-p53 interface. Alanine scanning experiments (replacement of wild type residues in p53 by alanine and then measuring the change in binding free-energy of p53 with MDM2) have determined the existence of three hot spots (main contributors to binding energy) in p53: PHE19, TRP23 and LEU26 [19]. Thus, we computationally replaced these residues by alanine in order to determine their impact on the stability of the C-HB of MDM2. First, we computationally mutated PHE19 to alanine in wild type p53 and we performed simulations within the complex MDM2-p53 (PDB: 1YCR). In other words, we retained all coordinates in the 1YCR structure but replaced the side chain of PHE19 by a methyl group, the side chain of ALA. Additionally, since in such PDB structure p53 is represented just by the helix that conforms its binding site (not the complete protein but just ten aminoacids, PDB: 1YCR) the position of the α carbons of such residues were simulated subjected to restraints in order to prevent the possible dismantling of the corresponding helix. We then studied the impact this replacement produced on the hydrogen bonds regarded as C-HBs in MDM2. From the five C-HBs (see Table 1) we found that four of them were not significantly perturbed, while the dynamics of HB 62–58 was enhanced. In fact, from the formation propensity of such HB we found that the PHE to ALA mutation in p53 increased the tendency to breakage of this HB in 103% as compared to the value it presented in the wild type complex (the fraction of time broken was less than 6% of the time, as shown in Table 1, while now increased to around 12%). We note that this labilization of the HB is significant in relative terms, while not in absolute value. This, however, can be explained in terms of the kind of computational experiment we are performing since we replace the side chain of the residue *within* the PDB of the wild type complex where the whole binding environment is already established and might be providing additional stabilization to the HB under study. This kind of simulation is much less time-demanding than a more reliable simulation of the alanine scanning process (in the experimental alanine scanning experiments, the p53 protein is first mutated and then subjected to association with MDM2). However, this computational procedure is free form the usual inaccuracies inherent to any simulation process of protein binding. We note also that we are not interested in estimating the energetics of the hot spot but to elucidate, qualitatively, the effect or impact the mutation exerts in the MDM2 C-HBs. In this sense, our results do in fact exhibit a clear tendency for HB destabilization since the HB 62–58 is softened in the mutated complex and its formation propensity falls to less than half the value it presented in the wild-type complex. In turn, the computational mutation of TRP23 to ALA showed an increase of 55% in the disruption tendency of the C-BHB 57–53 as compared to the wild-type situation. Finally, the mutation LEU 26 to ALA decreased the propensity formation of the C-HB 99–95 in 102% in comparison with wild-type tendency, such that in the mutated complex this interaction is now broken 35% of the time. These results indicate that the mutation of these rather large hydrophobic residues to alanine result in a labilization of certain C-HBs of the complex that now lack the stabilizing effects of the deleted side-chains (protection from the disruptive effect of water hydration [6–10]). Thus, even when applied under simplified simulation conditions that might inherently prevent it from attaining quantitative agreement, the dynamic analysis of HB propensity is indeed able to provide qualitative insights to help rationalize the results of alanine scanning experiments.

### Study of the binding of the disrupting molecule Nutlin-3a to MDM2

Nutlin-3a is a potent inhibitor of the MDM2-p53 interaction since it binds with high affinity to the p53 binding site of MDM2 protein [19, 20]. This disruptive drug mimics the action of p53 since it places hydrophobic moieties in the places where the protein locates the three hydrophobic residues (PHE19, TRP23 and LEU26) identified as hot spots [19, 20]. We studied the binding process of Nutlin3 to MDM2 by performing molecular dynamics simulations of the interaction of Nutlin-3a molecule with the MDM2 protein starting from its apo structure. For the apo protein we used the PDB 1Z1M deleting the first 25 residues in order to prevent form possible blockage of the p53 binding site. We also deleted the last ten residues in order to compare with the PDB for the complex MDM2-p53, PDB 1YCR, which lacks such residues (the C-HB 103–99 resides in such region, so we are not going to considered it in this analysis). This structure was then minimized and equilibrated in order to obtain constant Root Mean Squared Displacement values (for 50 ns). Then, we formed the complex by superposition of the coordinates of MDM2 in this apo structure and in the PDB in complex with Nutlin-3a (PDB: 4HG7). Finally, we produced the binding results, which were averaged over four different replicas, each of them of 20 ns length.

**Fig 1:**
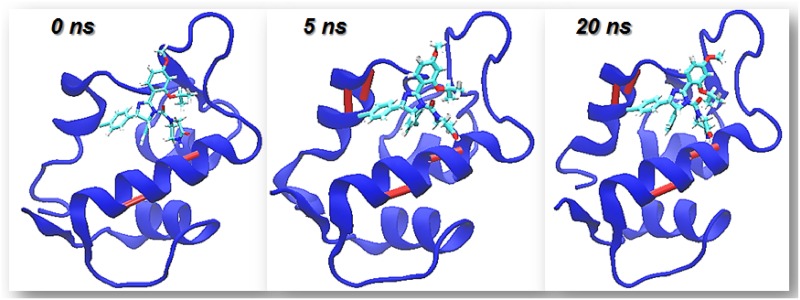
Different snapshots of the simulation of the interaction of MDM2 apo with Nutlin-3a illustrating the timescales of stabilization of the different C-HBs. If the C-HB is formed it is indicated by a bar colored in maroon.

Fig 1 shows the time evolution of the 3D structure of the MDM2 molecule during the binding process. From such picture we can learn that the binding site of MDM2 gets progressively structured in time as the C-HBs of the protein are intermolecularly stabilized by the drug. In particular, a helix that is mostly dismantled in the apo protein is well structured by the formation of the complex.

In turn, we also studied the dynamic formation propensity of the HBs identified as C-BHB (in the apo MDM2 protein) for the complex MDM2-Nutlin-3a. We started from the apo MDM2 protein and simulated the binding process of the small molecule. In Table 2 we show the stability of the different C-HBs after the complex is formed. We can see that the small molecule stabilizes all of the otherwise labile C-BHs (in the apo protein). In the case of HBs 99–95 and 100–96 this stabilization is only partial since they still retain certain lability.

**Table 2:** D-values: Fraction of time the C-HBs of MDM2 are broken for Nutlin-3a-MDM2 complex (D-values represent averages over four different replicas). The C-BHs are indicated by the numbers of the pair of residues involved in the hydrogen bond.

C HBs		MDM2apo- Nutlin-3a
		D
57	53	0.0475
62	58	0.06475
99	95	**0.31**
100	96	**0.33**

**Fig 2:**
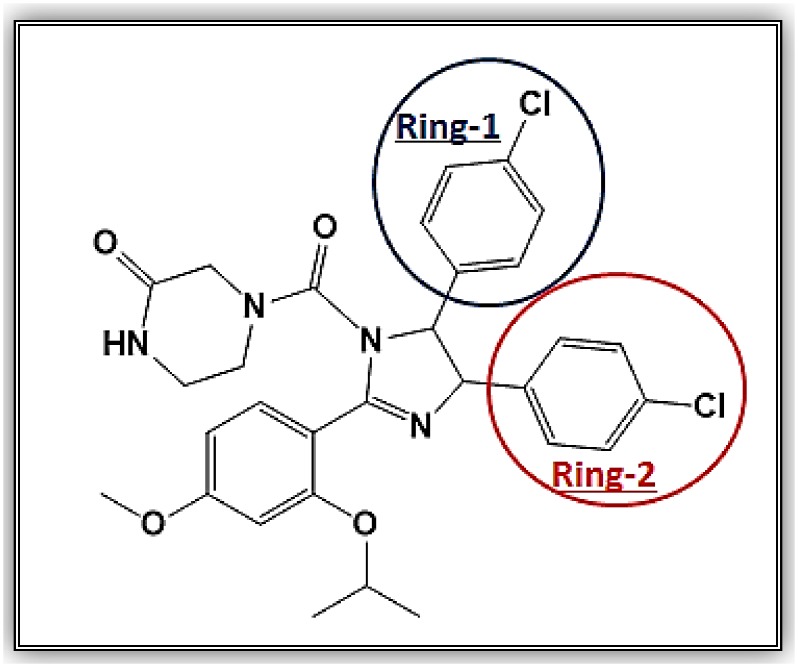
Nutlin3-a molecule and the two rings we detached in order to asses for their individual contribution to the binding process.

We also decided to dissect the contributions of the different groups of the molecule in this stabilizing effect. Thus, we conducted molecular dynamics simulations as the ones described above, but after detaching ring 1 or ring 2 in Nutlin-3a molecule as illustrated in Fig 2.

The results are displayed in Table 3. We can learn that the two rings of Nutlin-3a present different impacts on the stabilization of 62–58, 99–95 and 100–96 hydrogen bonds. Thus, by comparing the results of these two Nutlin-3a fragments with the effect of the complete molecule or with the effect of p53, we can envision the way this method could be useful for lead optimization in drug design: Lead compounds would perform a suboptimal C-HB quenching at different C-HBs and thus, the dynamic analysis of HB propensity could point out to regions of the molecule that should be substituted/improved in order to increase the binding affinity.

**Table 3:** D-values, that is, fraction of time the C-HBs of MDM2 are broken in the simulation of the complex of MDM2 with the two fragments of Nutlin-3a molecule. The four C-HBs of MDM2 are indicated by the numbers of the pair of residues involved in the HB interaction. Each fragment of Nutlin 3a is indicated by the ring we have deleted, as illustrated in Fig 2.

CBHBs		Without ring- 1	Without ring- 2
nRES	nRES	D	D
57	53	0.061	0.079
62	58	**0.49**	0.3425
99	95	0.229	**0.8745**
100	96	0.395	**0.567**

### Study of the binding of molecules with different affinity for MDM2

Finally, to also illustrate the potential of the backbone hydrogen bond propensity analysis for drug design, we also study the binding of a set of molecules with different affinities for MDM2 and perform the analysis of their C-BHB stabilization. This measure is then compared with traditional measures of binding affinity. The molecules under study are compound 14-b [22], nutlin-3a [20], compound 1-a [23] and compound DING-1a [24].

**Fig 3:**
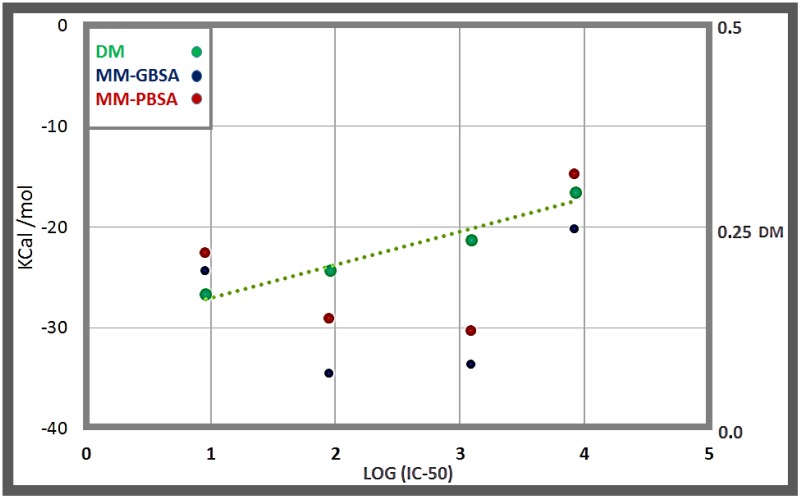
Correlation of the DM value with IC50 for different molecules that bind MDM2: compound 14-b [22], nutlin-3a [20], compound 1-a [23] and compound DING-1a [24], whose IC-50 are 9.2, 90, 1230 and 8400 nM, respectively. We also include the values of binding energy calculated by the MM-PBSA and MM-GBSA methods.

In all cases (please refer to the beginning of section 3.3 for the details where we introduce the case of Nutlin 3a), we formed the corresponding complexes by superposition of the coordinates of MDM2 in the structure of the apo protein and in the PDB in complex with the corresponding molecule (for the cases when the MDM2-molecule complexes are crystallized) and then produced the binding simulation runs (four replicas in each case). In the case of DING-1a (whose complex with MDM2 lacks a PDB structure), the starting configuration for the production runs was obtained by means of docking (Autodock 4.0). In all cases we obtained a value that we called DM, the average between the D-values of all the C-HBs (for each of the four disrupting molecules we also averaged over the 4 different replicas generated in each case). The fact that a PDB structure for the complex MDM2-DING-1a is lacking, does not represent any problem since, as we have already indicated D can be equivalently calculated as the fraction of time the C-HB is broken in the simulation of the complex (for the molecules where we have a PDB D is the difference between the PDB state-value = 1 and the fraction of time formed in the dynamics and, thus, also represents the fraction of time the interaction is broken in the simulations). Additionally, we compared such DM values with the corresponding binding energy measurements: GBSA/PBSA, evaluated for 1000 configurations equally spaced during a total timespan of 20 ns. The results are displayed in Table 4 and Fig 3. We can learn that the DM measure (that averages the quenching produced for the different C-HBs) displays a nice linear correlation with the experimental affinity values of the different molecules (measured by their corresponding IC50 values). In fact, the DM performs much better than the GBSA or PBSA measurements which display qualitative correlations with IC50 but with poorer quantitative performances. Thus, these results show that a method based on the analysis of hydrogen bond dynamic propensity is able to nicely discriminate between molecules with different binding potency.

**Table 4:** DM values for different p53-MDM2 disruptive molecules. We consider the molecules compound 14-b [22], nutlin-3a [20], compound 1-a [23] and compound DING-1a [24]. For each of the MDM2 C-HBs (indicated by the pair of residues comprised in the HB) we calculate the D-values and, by averaging such quantities, we provide the DM-value for the molecule.

Molecule		Comp- 14b	Nutlin-3a	comp- 1a	Comp-Ding-1a
IC-50 nM		9.2	90	1230	8400
C HBs		D	D	D	D
57	53	0.083	0.065	0.076	0.073
62	58	**0.317**	**0.314**	**0.328**	**0.368**
99	95	0.116	**0.338**	**0.442**	**0.494**
100	96	0.147	0.066	0.087	**0.236**
**DM**		**0.166**	**0.195**	**0.2329**	**0.292**

For completion, in S1–S6 Tables we provide the N-O distance of the MDM2 C-HBs for the different configurations of all systems studied in this work, from which the dynamic propensity matrices can be built.

## Conclusions

Even when the structure of a protein as recorded in the PDB provides us with invaluable information, certain aspects could be masked by the static picture of one or a few minimized configurations. In particular, dynamical information might be relevant for certain aspects related to protein binding and function. Specifically, we have shown that certain backbone hydrogen bonds (C-HB) in apo proteins present a dynamical behavior (a formation propensity in solution) that clearly differs from the state-value prescribed by the PDB structure and that, in turn, these labile hydrogen bonds are stabilized upon binding [11]. Thus, the analysis of backbone hydrogen bond dynamic propensity might be useful for protein binding and drug design strategies. In this sense, in this work we have rationalized the alanine scanning results for the complex p53-MDM2 in terms of this novel conceptual framework. Additionally, we have interpreted the binding to MDM2 of the disruptive molecule Nutiln3-a, also dissecting the contribution of different parts of the molecule as they stabilize C-HBs. Furthermore, we have shown that a measure rooted in this concept is able to successfully discriminate the affinity of different molecules for the MDM2 interface, even performing much better than traditional binding energy calculations. This method, could be useful not only to rationalize the optimal or suboptimal behavior of a given molecule at different regions (C-HBs) of the binding site, but might also enable re-engineering efforts to improve existing lead compounds. In this sense, our method relies on a single ingredient (the dynamic analysis of HB dynamic propensity) to asses binding affinity. Thus, it might complement existing methods by providing an additional operational principle to engineer better scaffolds.

## Supporting Information

S1 Table N-O distance for the different configurations of the simulation of MDM2 apo and MDM2-p53. We depict the N-O distance in Å for the different C-HBs (indicated by the pair of residues engaged in the HB) for all consecutive configurations of the MD run. Configurations are equally spaced and cover a time span of 40 ns.
(XLSX)

S2 Table N-O distance for the different configurations of the simulation of MDM2 in complex with Nutlin-3a. We depict the N-O distance in Å for the different C-HBs (indicated by the pair of residues engaged in the HB) for all consecutive configurations of 4 independent MD runs, each of 20 ns length.
(XLSX)

S3 Table N-O distance for the different configurations of the simulation of MDM2 in complex with two fragments of Nutlin-3a (detaching ring- 1 or ring- 2). We depict the N-O distance in Å for the different C-HBs (indicated by the pair of residues engaged in the HB) for all consecutive configurations of the MD run. Configurations are equally spaced and cover a time span of 40 ns.
(XLSX)

S4 Table N-O distance for the different configurations of the simulation of MDM2 in complex with comp- 14b. We depict the N-O distance in Å for the different C-HBs (indicated by the pair of residues engaged in the HB) for all consecutive configurations of 4 independent MD runs, each of 20 ns length.
(XLSX)

S5 Table N-O distance for the different configurations of the simulation of MDM2 in complex with comp- 1a. We depict the N-O distance in Å for the different C-HBs (indicated by the pair of residues engaged in the HB) for all consecutive configurations of 4 independent MD runs, each of 20 ns length.
(XLSX)

S6 Table N-O distance for the different configurations of the simulation of MDM2 in complex with comp-Ding-1a. We depict the N-O distance in Å for the different C-HBs (indicated by the pair of residues engaged in the HB) for all consecutive configurations of 4 independent MD runs, each of 20 ns length.
(XLSX)
